# American Medical Association

**Published:** 1884-10

**Authors:** Jno. S. Marshall


### AMERICAN MEDICAL ASSOCIATION.

### SECTION OF ORAL AND DENTAL SURGERY.

### MEETING AT WASHINGTON, MAY, 1884.

REPORTED FOR THE INDEPENDENT PRACTITIONER BY JNO. S. MARSHALL, M. D., SECRETARY OF THE SECTION.

(Continued from page 511.)

"Over-draught of Vital or Nervous Power, as Affecting General or Special Health;" by Dr. Jacob L. Williams, Boston, Mass.

SYNOPSIS.

The relations of reserve strength or nervous power to the general health and to the harmonious working of all the functions and faculties, every practitioner has constant occasion to observe. To him it is a great source of reliance for recovery from abnormal conditions and for continued health.

The diseases of different parts of the body have given rise to the various specialties in practice, all of which, when properly considered, have their relations to the general laws and forces controlling the system, and the question may be asked whether in some special departments these general laws are not often overlooked in the absorbing attention given to local disease.

It may be proper for the various specialties to elaborate and demonstrate each for itself the relations to which I have referred, but my present purpose is to make a few suggestions with regard to this matter as affecting some of the conditions of the oral cavity.

I say *reserve* nervous strength, for that is what must be drawn upon in any contest between health and disease. Temporary fortification with artificial tonics alone, gives no lasting victory.

No doubt a large proportion of the people in the artificially active life of our civilization are maintaining an appearance of fair health, while using up every day all the reserve nerve force they have; consequently are liable to succumb at the first extra demand made upon their reserve strength. This fact is also constantly made manifest in our observations of the teeth and mouth of individuals who, whether from a constitutional disposition to over-activity or from obligatory or voluntary overwork, are drawing too heavily upon the vital forces, for in no other way can some of the persistent abnormal conditions of these organs be accounted for.

Take the very common case of the business or professional man made dyspeptic from overwork. He is advised "to be careful of his diet," etc. This advice is followed, but he still keeps up his habits of overwork, and the diseased condition is not cured. Then rest is directed, and if taken in the right way improvement begins. Now, the idea is rational and seems capable of proof, that not only the gastric fluids but the secretions of other parts of the digestive system are abnormally affected by the same causes.

In cases such as the one just referred to, a greater or less tendency exists to unhealthy conditions of the oral secretions, which hold in solution fermentive material, and must as a chemical necessity sooner or later cause damage to the dental organs. This condition most commonly follows a continuous rather than a temporary over-strain, though I have seen cases of very rapid results from great nerve exhaustion.

Would suggest that the tendency to rapid decay of the teeth might be an aid in the diagnosis of obscure nervous affections.

This relationship of cause and effect I have repeatedly seen strikingly illustrated in young persons, who are more susceptible in this respect than adults, so that now I commonly expect to find more and greater activity of disease in the mouths of young scholars in the *spring* and *early* summer, after a winter season of unremitting application to study and exciting amusements, than in the autumn on their return from a summer vacation of leisure and rest.

Several cases illustrative of the above facts were described.

A similar condition of the mouth very commonly exists, as is well known, during pregnancy.

Another pathological condition in the mouth, which I have seen follow or accompany this exhaustion of vital force, is neuralgic pains at the roots of the teeth, particularly at the roots of the inferior central incisors, the pain in some cases extending along the line of both jaws, the teeth, however, all appearing healthy, and giving no indications of disease under the usual tests. When this disturbing cause is continuous for some time, I have noticed its liability to be followed by some degree of loosening and unsteadiness of the teeth, even when not accompanied with salivary calculus or any evidence of alveolar necrosis. This condition is no doubt sometimes taken for pyorrhœa alveolaris, and the heroic treatment adopted results as an aggravation of the malady.

DISCUSSION.

*Dr. T. C. Stellwagen,* Philadelphia, Pa.—I am always pleased to find an association of dental surgens willing to discuss other questions than the mechanical one of how to fill a tooth. It is refreshing to listen to some of the papers presented to this Section, and that of Dr. Williams has particularly pleased me. As a profession we have been inclined to overlook the importance of avoiding an estimate of the amount of nervous strain our patients are able to bear. There has been an undue interest shown in performing faultless mechanical operations, to the detriment of that most important question of the amount of the physical ability of the patient, which every surgeon should regard. Who has not seen a patient entirely exhausted after a long and tedious sitting for filling with gold, and remain prostrated for several days. Many indeed are utterly unable to endure such strain upon their nervous system. For such sufferers plastic filling materials have served an excellent purpose, and without them many teeth would have been lost.

This paper has also recalled some observations that I have made upon "Climatic influences, and their expression in the oral cavity." I have not infrequently seen cases in which there were rapid deteriorations of dental tissue, probably the result of climatic influences, as was apparently proved by the arrestation of the pathological conditions following a change to the air and method of living in the mountains or upon the sea-shore.

(Two cases were described which seemed to aid in substantiating the views advanced by the speaker.—*Reporter.*)

He also spoke of the importance of examinations as to the condition of the dentine to determine the progress of certain chronic or tedious diseases, and thought the removal of a filling to test the density, etc., of the dentine underneath would often prove of valuable assistance to general diagnosis and prognosis.

*Dr. Marshall*—I hope Dr. Stellwagen will continue his observations in this direction, for they are certainly interesting, and I am sure the Section would be glad to listen to a paper upon this subject at any time. Perhaps Dr. Stellwagen will be prepared to do so next year.

*Dr. Stellwagen*—I cannot promise for the next meeting, but may be able to at some future day.

*Dr. Friedericks*—I am very glad that Dr. Williams has called attention to this matter. It is worthy of more thought than has been spent upon it by the profession in general. I distinctly remember one patient, a little girl, who suffered from cerebro-spinal fever, induced by the nervous strain incident to a long and tedious dental operation. The child recovered, but was an invalid for months. Such experiences ought to teach us a valuable lesson.

*Dr. Allport*—In my early practice I was in the habit of paying little regard to the condition of my patients when presenting themselves for dental operations, but latterly have seldom subjected a child or a frail adult to long, tedious operations, and then only under the most urgent necessity. I prefer to use some plastic filling material, like the phosphate of zinc, or gutta percha, and occasionally amalgam.

We have been apt to laugh at the operators known as "new departure" men, but we must confess that plastic operations have done very much to alleviate the sufferings and preserve the teeth of individuals just mentioned. I think it much better to use plastic fillings in such cases, and renew them as occasion requires until the health or nervous system is sufficiently strong to endure the strain of a permanent operation.

